# Efficacy of Metribuzin Doses on Physiological, Growth, and Yield Characteristics of Wheat and Its Associated Weeds

**DOI:** 10.3389/fpls.2022.866793

**Published:** 2022-05-02

**Authors:** Muhammad Mansoor Javaid, Athar Mahmood, Muhammad Izhar Naeem Bhatti, Hasnain Waheed, Kotb Attia, Ahsan Aziz, Muhammad Ather Nadeem, Naeem Khan, Abdullah A. Al-Doss, Sajid Fiaz, Xiukang Wang

**Affiliations:** ^1^Department of Agronomy, College of Agriculture, University of Sargodha, Sargodha, Pakistan; ^2^Department of Agronomy, University of Agriculture, Faisalabad, Pakistan; ^3^Department of Biochemistry, College of Science, King Saud University, Riyadh, Saudi Arabia; ^4^Department of Agronomy, Institute of Food and Agricultural Sciences, University of Florida, Gainesville, FL, United States; ^5^Biotechnology Lab, Plant Production Department, College of Food and Agriculture Sciences, King Saud University, Riyadh, Saudi Arabia; ^6^Department of Plant Breeding and Genetics, The University of Haripur, Haripur, Pakistan; ^7^College of Life Sciences, Yan’an University, Yan’an, China

**Keywords:** adjuvants, herbicide doses, photosynthetic activity, triticum aestivum, weeds control

## Abstract

Weeds cause a serious constraint to wheat productivity. Chemical weed control is considered the most effective method to control weeds; however, a suitable dose and combination of herbicide with adjuvants play a vital role in controlling weeds and producing maximum wheat production. A field study was conducted to investigate the effectiveness of various doses of metribuzin alone or in combination with adjuvants [Bio-power (alkyl ether sulfates and sodium salts) and Ad-500 (fatty alcohol ethoxylate)] on the growth and yield of wheat and its associated weeds. Metribuzin at 175, 140, and 105 g a.i ha^–1^, each in combination with adjuvants (Bio-power or Ad-500) at 400 ml ha^–1^, were sprayed. A weedy check was also included as a control treatment. The wheat crop was infested with *Fumaria indica*, *Melilotus indica*, *Anagallis arvensis*, and *Phalaris minor*, and metribuzin with or without adjuvant was sprayed at two- to four-leaf stage of the weeds. The photosynthetic activity, weed population of each weed, and biomass of each weed was significantly affected by all herbicides along with the adjuvant. However, maximum inhibition of tested weeds was observed where metribuzin at 175 g a.i ha^–1^ + Bio-power at 400 ml ha^–1^ were sprayed. Metribuzin sprayed at 175 g a.i ha^–1^ with or without Bio-power recorded a maximum 1,000-grain weight, biological yield, and grain yield. Conclusively, metribuzin sprayed at 175 g a.i ha^–1^ + Bio-power at 400 at ml ha^–1^ have the potential to improve wheat yield by inhibiting weed growth, and Bio-power was superior to Ad-500 in improving the efficacy of metribuzin against weeds of wheat crop.

## Introduction

Wheat is one of the most important crops among the world’s cereals (Aula et al., 2019). Wheat production is restricted by several factors in which severe weed interference is the most obstructing factor (Scavo and Mauromicale, 2020; Gandía et al., 2021; Hussain et al., 2021). Unrestricted weed growth throughout the life cycle of wheat leads to a 20%–40% decline in yield (Ahmad and Shaikh, 2003). Losses due to weeds can be managed by different approaches, but chemical weed control is the most improved method (Mehmood et al., 2018; MacLaren et al., 2020). However, the excessive use of herbicides may cause environmental and human health hazards (Hasanuzzaman et al., 2020). The wise use or application of herbicides also helps to overcome the resistance and environmental pollution problems (Alister and Kogan, 2006; Beaudegnies et al., 2009). Due to increasing concerns about food safety, environmental integrity, and weed resistance, it is imperative to develop approaches that could enhance herbicide efficacy at a lower dose. In cereal, particularly in wheat, different herbicides are utilized with the mode of action of photosynthesis inhibition (Dodge, 2019) by interrupting the normal functioning of chlorophyll and photosystems I and II by protein D-1 binding of quinine in the thylakoid membrane and seizes electron transport chain (Soltani et al., 2006; Naseer-ud-din et al., 2011).

Metribuzin, an effective postemergence triazine group herbicide with the mode of action of photosynthesis inhibition (Shah et al., 2009; Štěpánová et al., 2012), is often applied to restrict the broadleaf and annual grass weeds in wheat, potatoes, and tomatoes (Majumdar and Singh, 2007). The efficiency of herbicides that are used after emergence can be enhanced by adding adjuvants (Javaid et al., 2012, 2020; Moraes et al., 2021). Adjuvants help to reduce herbicide dose and improve its biological activity by altering the surface tension of spray solution, increasing absorption, translocation rate, effect on atomization, and enhanced penetration (Hamouda et al., 2021). Some adjuvants completely alter the chemical composition of herbicide or even cover the plant surface by keeping the herbicide in contact with plant tissue and hence increase the herbicide penetration and binding ability to kill the target plant without damaging the actual crop (Dubovik et al., 2020). Several studies have demonstrated that adjuvant’s efficacy is dependent on the herbicide being used and the characteristics of the target weed (Javaid and Tanveer, 2013). Other studies showed that the mixing of adjuvants in herbicides caused an increase, a decrease, or no change in the herbicide efficacy (Ferreira et al., 2020). It is documented that identification of a suitable adjuvant to be added in a specific herbicide for the control of a particular weed is necessary to enhance herbicide efficacy (Penner, 2000; Azza et al., 2020). In the present scenario of increasing concerns environment, there is a dire need to optimize the herbicide efficacy without affecting crop productivity. Thus, the objectives of this research were to determine the effect of the addition of adjuvants (Bio-power or Ad-500) on the efficacy of metribuzin at recommended or reduced doses for producing wheat and controlling wheat weeds.

## Materials and Methods

### Field Studies

In the winter season of 2013–2014 and 2014–2015, field trials were executed to assess the influence of various metribuzin dosed alone and with the mixture of adjuvant on wheat and its allied weeds. The experiment was laid out at the Agronomic Research Area, College of Agriculture, University of Sargodha, Sargodha (latitude 31.41°N, longitude 74.17°E, and altitude 194.4 m). Experimental soil was sandy to clay loam with pH 8, organic matter 0.89%, nitrogen 0.51%, phosphorus 11 ppm, and potash 109 ppm. Plot size was 1.5 m × 3.5 m, and 25-cm row spacing was maintained. Punjab-2011 wheat cultivar was grown on November 27, 2013, and November 30, 2014, at a seed rate of 100 kg ha^–1^. Nitrogen (urea), phosphorus (diammonium phosphate), and potassium (sulfate of potash) were applied at the rates of 50, 34, and 25 kg ha^–1^, respectively. The experimental area was maintained with conventional tillage, with crop rotation of wheat sorghum [*Sogrhum bicolor* (L.) wheat], and contained the dense population of *Fumaria indica*, *Melilotus indica*, *Anagallis arvensis*, and *Phalaris minor*.

### Herbicide Treatments and Application

Different doses of metribuzin with or without adjuvants were sprayed on wheat crops. Two adjuvants, namely, Bio-power (alkyl ether sulfates and sodium salts) and Ad-500 (fatty alcohol ethoxylate) were mixed in the metribuzin according to the treatments: metribuzin at 175, 140, and 105 g a.i ha^–1^ alone, metribuzin at 175 g a.i ha^–1^ + Ad-500 at 400 ml ha^–1^, metribuzin at 140 g a.i ha^–1^ + Ad-500 at 400 ml ha^–1^, metribuzin at 105 g a.i ha^–1^ + Ad-500 at 400 ml ha^–1^, metribuzin at 175 g a.i ha^–1^ + Bio-power at 400 ml ha^–1^, metribuzin at 140 g a.i ha^–1^ + Bio-power at 400 ml ha^–1^, and metribuzin at 105 g a.i ha^–1^ + Bio-power at 400 ml ha^–1^. Plots that contained weeds other than *F. indica*, *M. indica*, *A. arvensis*, and *P. minor* were removed by hand before treatment application. Spray composition was prepared in a plastic bottle by adding metribuzin with or without adjuvants containing tap water. Each dose of metribuzin without or with adjuvants was prepared separately in a plastic bottle, and the bottle was washed thoroughly before the preparation of the next treatment of herbicide composition. All herbicide treatments were applied with a backpack sprayer equipped with a flat-fan nozzle (Teejet 8002E nozzle, Spraying Systems Company, Wheaton, IL 60118) calibrated to deliver 250 L ha^–1^ at a speed of 3.2 km h^–1^. The treatments were applied at the two- to four-leaf stage of weeds.

### Data Collection and Analysis

Data pertaining to growth (plant height, spike length, and biological yield) and yield traits (number of spike-bearing and non-spike-bearing tillers, 1,000-grain weight, number of grains per spike, and grain yield) were recorded. The spike-bearing and non-spike-bearing tillers were counted in an area of 1 m^2^ when spikes fully emerged from the flag leaf. Ten spikes were harvested from each plot and hand trashed to record grain number per spike. A total of 1,000 grains were collected from each plot and weight at 12% moisture contents. The middle four rows of the wheat from each plot were harvested and allowed to sun-dry for 5 days and recorded the biological yield. The grain yield was adjusted to 12% moisture contents.

At 21 days after herbicide application, the surviving plants of *F. indica*, *M. indica*, *A. arvensis*, or *P. minor* were counted. Evaluation of tested weeds control was also done at maturity of the wheat crop, and weeds count was done 7 days before wheat harvesting. Weeds plant height was measured on random plants 7 days before wheat harvest. The above-ground biomass of each weed was determined by harvesting plants from an area of 1 m^2^ 3 days before wheat harvest, and fresh weight was recorded. The above-ground of each weed was then oven-dried at 72°C for 48 h to obtain the dry weight of each weed.

The gas exchange parameters such as net photosynthetic rate, transpiration rate, and stomatal conductance of wheat and its associated weeds were recorded with a portable infrared gas analyzer (CI-340 Portable Photosynthesis System, CID Biosciences, United States) at three stages (3, 10, and 17 days after herbicide spray). The adjustments of the photosynthesis system were as follows: mass flow rate of 0.33 mol m^–2^ s^–1^, atmospheric pressure of 99.5 kPa, photosynthetically active radiation at leaf surface of up to 1,393 μmol m^–2^ s^–1^, the water vapor pressure at the outlet of leaf chamber ranging from 1.7 to 2.4 kPa, and the temperature of the ambient air in the leaf chamber ranging from 23 to 34°C. The photosynthesis system reading was recorded between 9:00 and 11:00 a.m. on fully expanded leaves, wheat, or weeds.

### Statistical Analysis

The trial was conducted under randomized complete block design (RCBD) with four repetitions. Data collected in both years were non-significant, and the data were pooled over the years. The response variables were analyzed with the software Statistix 10© (Analytical Software, Tallahassee, FL). An analysis of variance (ANOVA) was performed to identify differences between individual treatments. The model structure of ANOVA was


Y=ijμ+T+i€ij

where Y_*ij*_ is the observed response variable, μ is the overall mean, T_*i*_ is the explanatory variable, and €_*ij*_ is the error. The significant differences among treatment means were identified using Fisher’s LSD at *P* < 0.05 (Steel et al., 1997). The graphical demonstration of the data was performed using Sigma plot 2008 (version 11.0). The average of both years’ data was presented for each parameter.

## Results

### Growth and Yield Attributes of Wheat

Spike-bearing and non-spike-bearing tillers of wheat were considerably influenced by all doses of applied herbicide (Table 1). Among the treatments, maximum productive tillers (389.5 m^–2^) were noted with metribuzin at 105 g a.i ha^–1^ + Bio-power at 400 ml ha^–1^. The lowest spike-bearing tillers (326.2 m^–2^) were observed under weedy check. In contrast, the lowest non-spike-bearing tillers were counted where metribuzin at 175 g a.i ha^–1^ + Bio-power at 400 ml ha^–1^ were sprayed while maximum non-spike-bearing tillers were observed with control treatment. The data presented in Table 1 depicted that extreme decline in wheat height was noted under weedy check but the taller wheat plant (90.5 cm) was measured with metribuzin at 140 g a.i ha^–1^ + Bio-power at 400 ml ha^–1^. Metribuzin sprayed with or without adjuvants imparted a positive impact on grains per spike and length of the spike (Table 1).

**TABLE 1:** Influence of metribuzin alone and with adjuvants on number of productive tillers (m^–2^), number of non-productive tillers (m^–2^), plant height (cm), and number of grains per spike of wheat (average of 2 years’ data).

Treatments	Number of Productive tillers (m^–2^)	Number of non-productive tillers (m^–2^)	Plant height (cm)	Spike length (cm)
T_1_	326.2 c	35.2 ab	81.8 c	9.43 b
T_2_	369.0 abc	24.7 bc	90.9 ab	10.0 ab
T_3_	377.0 ab	24.5 bc	91.7 ab	10.2 ab
T_4_	337.0 bc	22.2 c	89.2 b	9.9 ab
T_5_	350.7 abc	25.7 abc	90.0 ab	10.2 ab
T_6_	379.7 ab	34.2 ab	91.5 ab	9.88 ab
T_7_	340.0 abc	37.5 a	90.3 ab	9.97 ab
T_8_	374.2 abc	19.2 c	88.2 b	9.7 b
T_9_	365.2 abc	23.7 bc	95.5 a	10.8 a
T_10_	389.5 a	28.2 abc	90.6 ab	10.1 ab
HSD (0.05)	50.48	11.86	6.18	1.08

*Means sharing the same letter in a column did not differ from each other at a 5% (0.05) probability level. T_1_, weedy check; T_2_, metribuzin at 175 g a.i ha^–1^; T_3_, metribuzin at 140 g a.i ha^–1^; T_4_, metribuzin at 105 g a.i ha^–1^; T_5_, metribuzin at 175 g a.i ha^–1^ + Ad-500 at 400 ml ha^–1^; T_6_, metribuzin at 140 g a.i ha^–1^ + Ad-500 at 400 ml ha^–1^; T_7_, metribuzin at 105 g a.i ha^–1^ + Ad-500 at 400 ml ha^–1^; T_8_, metribuzin at 175 g a.i ha^–1^ + Bio-power at 400 ml ha^–1^; T_9_, metribuzin at 140 g a.i ha^–1^ + Bio-power at 400 ml ha^–1^; T_10_, metribuzin at 105 g a.i ha^–1^ + Bio-power at 400 ml ha^–1^.*

Spike length (10.86 cm) of wheat was maximum when metribuzin at 140 g a.i ha^–1^ + Bio-power at 400 ml ha^–1^ were sprayed. The minimum length of the wheat spike (9.43 cm) was recorded where no herbicide was sprayed. However, results demonstrated that maximum spike length was noted where metribuzin was applied with adjuvants at 140 g a.i ha^–1^ + Bio-power at 400 ml ha^–1^ as compared with the plots where metribuzin alone was sprayed. Metribuzin at 105 g a.i ha^–1^ + Bio-power at 400 ml ha^–1^ produced the highest grains per spike (Table 2).

**TABLE 2:** Influence of metribuzin alone and with adjuvants on a number of grains per spike, 1,000-grain weight (g), biological yield (kg ha^–1^), and grain yield (kg ha^–1^) of wheat (average of 2 years’ data).

Treatments	Number of grains per spike	1000-grain weight (g)	Biological yield (kg ha^–1^)	Grain yield (kg ha^–1^)
T_1_	82.7 bc	40.0 abcd	b	1.39 cd
T_2_	82.5 bc	42.2 abc	ab	1.40 cd
T_3_	91.2 a	38.2 cd	9.52 ab	3.66 abc
T_4_	88.0 abc	39.5 bcd	9.44 ab	3.24 cd
T_5_	91.2 a	40.7 abcd	9.98 ab	3.77 ab
T_6_	81.0 c	43.0 ab	10.35 ab	3.54 bcd
T_7_	86.7 abc	38.0 d	8.64 ab	3.47 bcd
T_8_	86.5 abc	44.0 a	10.86 a	3.91 a
T_9_	90.2 ab	42.2 abc	10.49 ab	3.33 cd
T_10_	93.2 a	39.0 bcd	9.70 ab	3.38 cd
HSD (0.05)	7.77	2.40	0.971	0.293

*Means sharing the same letter in a column did not differ from each other at a 5% (0.05) probability level. T_1_, weedy check; T_2_, metribuzin at 175 g a.i ha^–1^; T_3_, metribuzin at 140 g a.i ha^–1^; T_4_, metribuzin at 105 g a.i ha^–1^; T_5_, metribuzin at 175 g a.i ha^–1^ + Ad-500 at 400 ml ha^–1^; T_6_, metribuzin at 140 g a.i ha^–1^ + Ad-500 at 400 ml ha^–1^; T_7_, metribuzin at 105 g a.i ha^–1^ + Ad-500 at 400 ml ha^–1^; T_8_, metribuzin at 175 g a.i ha^–1^ + Bio-power at 400 ml ha^–1^; T_9_, metribuzin at 140 g a.i ha^–1^ + Bio-power at 400 ml ha^–1^; T_10_, metribuzin at 105 g a.i ha^–1^ + Bio-power at 400 ml ha^–1^.*

The lowermost grains per spike (81.0) were noted with metribuzin at 140 g a.iha^–1^ + Ad-500 at 400 ml ha^–1^. The effect of metribuzin with or without adjuvants on 1,000-grain weight is exhibited in Table 2. All herbicide treatments produce more 1,000-grain weight than weedy check. Data exposed that a higher 1,000-grain weight of wheat (44 g) was weighed in plots where metribuzin at 175 g a.i ha^–1^ + Bio-power at 400 ml ha^–1^ were sprayed followed by metribuzin at 175 g a.iha^–1^ + Ad-500 at 400 ml ha^–1^. The lowest 1,000-grain weight of wheat (38.0 g) was noted under weedy check. Table 2 showed that the impact of all herbicide treatments was significant on the biological yield of wheat. Metribuzin at 175 g a.i/ha^–1^ + Bio-power at 400 ml ha^–1^ resulted in the highest biological yield followed by metribuzin at 140 g a.i ha^–1^ with both the adjuvants. The lowest value of biological yield was recorded from plots where no herbicide was sprayed. Wheat yield was varied under various applications of metribuzin without or with adjuvants. The application of metribuzin at 175 g a.i ha^–1^ with both adjuvants markedly enhanced the wheat yield over a weedy check. The addition of the Bio-power in metribuzin is more effective than that of Ad-500 addition because incorporation of Bio-power at 400 ml ha^–1^ with metribuzin (175 g a.i ha^–1^) showed maximum grain yield in comparison with all other treatments.

### Physiological Parameters of Wheat

**FIGURE 1:**
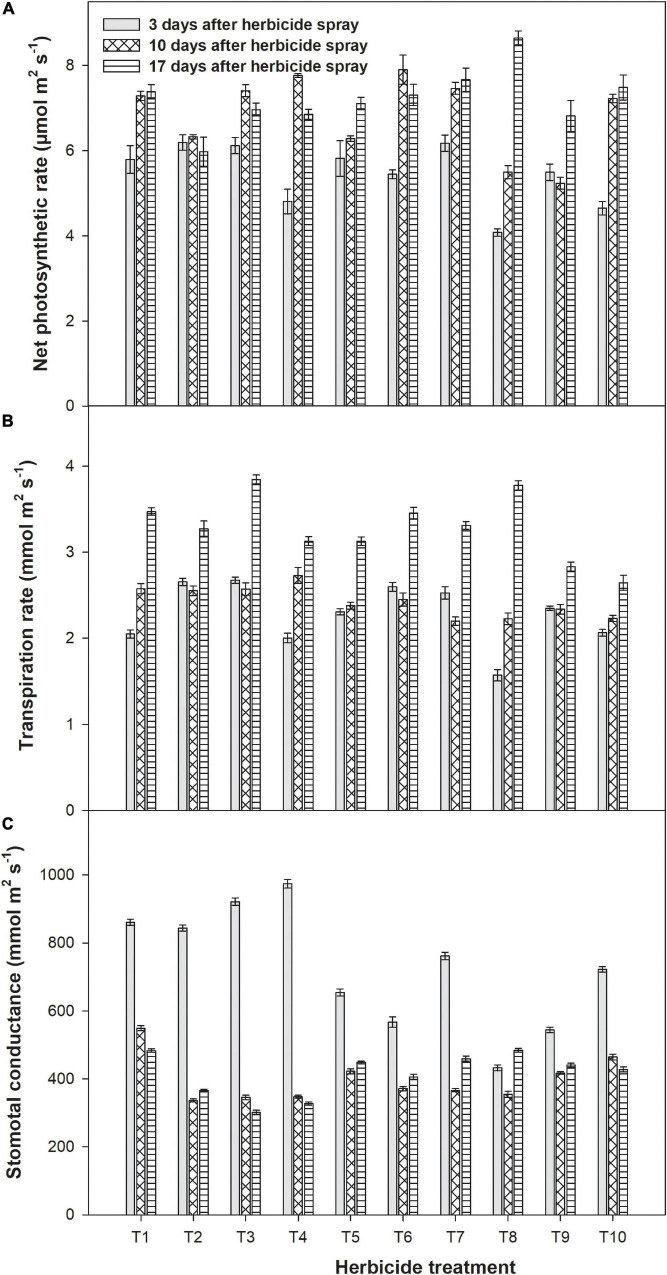
Impact of metribuzin with and without adjuvants on net photosynthetic rate **(A)**, transpiration rate **(B)**, and stomatal conductance **(C)** of wheat at 3, 10, and 17 days after herbicide spray (average of 2 years’ data). T_1_, weedy check; T_2_, metribuzin at 175 g a.i. ha^–1^; T_3_, metribuzin at 140 g a.i. ha^–1^; T_4_, metribuzin at 105 g a.i. ha^–1^; T_5_, metribuzin at 175 g a.i. ha^–1^ + Ad-500 at 400 ml ha^–1^; T_6_, metribuzin at 140 g a.i. ha^–1^ + Ad-500 at 400 ml ha^–1^; T_7_, metribuzin at 105 g a.i. ha^–1^ + Ad-500 at 400 ml ha^–1^; T_8_, metribuzin at 175 g a.i. ha^–1^ + Bio-power at 400 ml ha^–1^; T_9_, metribuzin at 140 g a.i. ha^–1^ + Bio-power at 400 ml ha^–1^; T_10_, metribuzin at 105 g a.i. ha^–1^ + Bio-power at 400 ml ha^–1^.

The net photosynthetic rate of wheat was recorded at 3, 10, and 17 days after metribuzin application alone or with adjuvants is shown in Figure 1. Results revealed that the net photosynthetic rate was higher at three stages (3, 10, and 17 days after spray) in weedy check plots. However, among all herbicides treatments, the lowest net photosynthetic rate of wheat (4.07 μmol m^2^ s^–1^) was observed with the application of metribuzin at 175 g a.i ha^–1^ + Bio-power at 400 ml ha^–1^ at 3 days after herbicide application followed by 10 and 17 days after herbicide application under the same treatment. In the case of transpiration rate, weedy check plots depicted maximum transpiration rate at three stages with all the herbicide treatment. Figure 1 depicted a linear increase in transpiration rate along with the growth of the wheat crop. After weedy check, a reduced dose of metribuzin (140 g a.i ha^–1^) without adjuvant showed highest transpiration rate (3.84 mmol m^2^ s^–1^) of wheat at the third stage of measurement. Whereas, the transpiration rate (2.17 mmol m^2^ s^–1^) was reduced with metribuzin at 175 g a.i ha^–1^ + Ad-500 at 400 ml ha^–1^ after 3 days of spray. The stomatal conductance of wheat was reduced with the application of herbicide. However, stomatal conductance was improved significantly when measurements were noted after 17 days after spraying herbicide as compared with other stages (Figure 1). Maximum stomatal conductance (1,260.5 mmol m^2^ s^–1^) was observed in the weedy check. However, metribuzin application at 175 g a.i ha^–1^ + Ad-500 at 400 ml ha^–1^ depicted the least stomatal conductance of wheat at all intervals of observation (Figure 1).

### Growth and Yield Parameters of Weeds

*Fumaria indica*, *M. indica*, *A. arvensis*, and *P. minor* were the weed species observed in the experimental area. Metribuzin alone or with adjuvants imparted significant impact on all tested weeds at 21 days after herbicide spray (Table 3). Results indicated that the maximum number of *M. indica* (58 m^–2^), *A. arvensis* (27.5 m^–2^), *P. minor* (7 m^–2^), and *F. indica* (7.5 m^–2^) were measured under weedy check treatment. Among all treatments of herbicide, metribuzin at 175 g a.i ha^–1^ + Ad-500 at 400 ml ha^–1^ recorded the minimum number of *A. arvensis*, *P. minor*, and *F. indica* weed plants. However, the minimum number of *M. indica* was documented with metribuzin application at 175 g a.i ha^–1^ + Bio-power at 400 ml ha^–1^ (Table 3).

**TABLE 3:** Influence of metribuzin alone and with adjuvants on a number of weeds (m^–2^) 21 days after herbicide application and at the harvest of wheat (average of 2 years’ data).

	Number of weeds 21 days after treatment				Number of weeds at crop harvest			
Treatments	*Melilotus indica* L.	*Anagallis arvesis* L.	*Phalaris minor* L.	*Fumaria indica* L.	*Melilotus indica* L.	*Anagallis arvesis* L.	*Phalaris minor* L.	*Fumaria indica* L.
T_1_	57.5 a	27.5 a	7.2 a	7.5 a	52.2 a	24.7 a	5.6 a	6.5 a
T_2_	8 bc	10.2 bc	2.5 bc	2.2 d	4.2 b	1.2 b	3.6 ab	4.0 b
T_3_	7.5 bc	12.0 b	4.0 b	3.5 bcd	4.0 b	1.5 b	4.0 ab	3.5 b
T_4_	8.5 b	7.5 cde	4.2 b	4.5 bc	4.0 b	2.5 b	3.6 ab	3.0 b
T_5_	4.0 de	6.5 def	3.0 bc	2.7 cd	1.5 bc	1.0 b	3.0 b	3.3 b
T_6_	6.5 bcd	7.2 cde	2.7 bc	2.7 cd	3.5 bc	2.2 b	3.3 ab	2.6 b
T_7_	6.0 bcd	7.5 cde	3.7 bc	4.7 b	1.2 bc	2.2 b	3.5 ab	3.0 b
T_8_	5.7 cd	4.2 ef	3.0 bc	2.7 cd	2.2 bc	1.7 b	2.3 b	4.0 b
T_9_	3.0 e	3.7 f	2.0 c	2.0 d	0.5 c	2.0 b	3.3 ab	2.0 b
T_10_	2.2 e	8.7 bc	3.2 bc	4.2 bc	1.0 bc	2.7 b	3.5 ab	3.0 b
HSD (0.05)	2.52	3.27	1.79	1.90	3.43	4.51	2.65	2.32

*Means sharing the same letter in a column did not differ from each other at a 5% (0.05) probability level. DAT, days after treatments; T_1_, weedy check; T_2_, metribuzin at 175 g a.i ha^–1^; T_3_, metribuzin at 140 g a.i ha^–1^; T_4_, metribuzin at 105 g a.i ha^–1^; T_5_, metribuzin at 175 g a.i ha^–1^ + Ad-500 at 400 ml ha^–1^; T_6_, metribuzin at 140 g a.i ha^–1^ + Ad-500 at 400 ml ha^–1^; T_7_, metribuzin at 105 g a.i ha^–1^ + Ad-500 at 400 ml ha^–1^; T_8_, metribuzin at 175 g a.i ha^–1^ + Bio-power at 400 ml ha^–1^; T_9_, metribuzin at 140 g a.i ha^–1^ + Bio-power at 400 ml ha^–1^; T_10_, metribuzin at 105 g a.i ha^–1^ + Bio-power at 400 ml ha^–1^.*

At the harvest stage, all herbicide treatments exerted a substantial impact on a number of weeds of all four species. Table 3 shows that the highest number of all weed species was observed under weedy check treatment. The minimum number of *M. indica* (0.5 m^–2^) and *F. indica* (2.0 m^–2^) were noted with metribuzin at 140 g a.i ha^–1^ + Bio-power at 400 ml ha^–1^. *A. arvensis* and *P. minor* weeds were minimal in plots where metribuzin at 175 g a.i ha^–1^ + Ad-500 at 400 ml ha^–1^ were sprayed. Though, metribuzin at an abridged dose (105 g a.i ha^–1^) + Bio-power and Ad-500 each at 400 ml ha^–1^ recorded the lowest number of *M. indica*, *F. indica*, and *P. minor* at the crop harvest stage as compared with those plots where metribuzin at 175 g a.i ha^–1^ without adjuvants was applied.

The fresh and dry weight of all tested weeds reduced significantly with all doses of metribuzin alone or with adjuvant as compared with control (weedy check) (Table 4). All four weeds produced the highest fresh and dry weight in control. Metribuzin at 175 g a.i ha^–1^ + Bio-power at 400 ml ha^–1^ suppressed the tested weeds at the maximal level and produced 37.5, 199.7, 17.2, and 27.5 g fresh weight of *M. indica*, *P. minor*, *F. indica*, and *A. arvensis*, respectively. In the case of the dry weight of weeds, the same dose of metribuzin with adjuvant resulted in minimal dry weight of tested weeds.

**TABLE 4:** Influence of metribuzin alone and with adjuvants on fresh and dry weight (g) of weeds growing in wheat (average of 2 years’ data).

	Fresh weight (gm^–2^)				Dry weight (gm^–2^)			
Treatments	*Melilotus indica* L.	*Anagallis arvesis* L.	*Phalaris minor* L.	*Fumaria indica* L.	*Melilotus indica* L.	*Anagallis arvesis* L.	*Phalaris minor* L.	*Fumaria indica* L.
T_1_	160.0 a	121.2 a	293.7 a	96.2 a	44.0 a	23.7 a	61.5 a	23.7 a
T_2_	40.5 cde	30.5 de	217.5 f	17.5 c	16.5 cd	8.7 bc	44.7 d	8.5 cd
T_3_	42.5 bc	32.6 c	236.2 e	22.5 bc	18.5 bc	10.0 bc	51.5 c	8.4 cd
T_4_	44.5 b	34.5 b	258.7 c	24.5 b	20.5 b	11.5 b	57.2 ab	12.5 b
T_5_	38.5 ef	28.5 fg	249.0 cd	18.5 c	14.5 de	8.5 c	49.5cd	8.5 cd
T_6_	41.0 cde	30.4 de	205.7 g	20.5 bc	16.5 cd	9.5 bc	55.0 bc	9.5 cd
T_7_	42.0 bcd	31.5 cd	240.7 de	21.5 bc	18.5 bc	10.5 bc	45.5 d	10.5 bc
T_8_	37.5 f	27.5 g	199.7 g	17.2 c	12.5 e	8.0 c	37.5 e	7.5 d
T_9_	38.5 ef	28.5 fg	273.7 b	18.5 c	14.5 de	9.0 bc	58.0 ab	8.5 cd
T_10_	39.5 def	29.5 ef	260.2 c	19.5 bc	16.5 cd	10.0 bc	49.7 cd	9.4 cd
HSD (0.05)	2.84	1.92	11.37	5.54	2.39	2.76	5.63	2.82

*Means sharing the same letter in a column did not differ from each other at a 5% (0.05) probability level. T_1_, weedy check; T_2_, metribuzin at 175 g a.i ha^–1^; T_3_, metribuzin at 140 g a.i ha^–1^; T_4_, metribuzin at 105 g a.i ha^–1^; T_5_, metribuzin at 175 g a.i ha^–1^ + Ad-500 at 400 ml ha^–1^; T_6_, metribuzin at 140 g a.i ha^–1^ + Ad-500 at 400 ml ha^–1^; T_7_, metribuzin at 105 g a.i ha^–1^ + Ad-500 at 400 ml ha^–1^; T_8_, metribuzin at 175 g a.i ha^–1^ + Bio-power at 400 ml ha^–1^; T_9_, metribuzin at 140 g a.i ha^–1^ + Bio-power at 400 ml ha^–1^; T_10_, metribuzin at 105 g a.i ha^–1^ + Bio-power at 400 ml ha^–1^.*

All applications of metribuzin alone or with adjuvants significantly suppressed the height of all four weed species present in wheat crop over the weedy check (Table 5). The maximum plant height of *M. indica*, *A. arvensis*, *P. minor*, and *F. indica* was recorded in the weedy check, whereas metribuzin at 140 g a.i ha^–1^ + Bio-power at 400 ml ha^–1^ recorded the height of 20, 1.5, and 46.9 cm of *M. indica*, *A. arvensis*, and *P. minor*, respectively, among all the treatments. Minimum height (16.3 cm) of *F. indica* was documented with metribuzin at 175 g a.i ha^–1^ + Bio-power at 400 ml ha^–1^.

**TABLE 5:** Influence of metribuzin alone and with adjuvants on plant height (cm) of weeds growing in wheat (average of 2 years’ data).

	Plant height (cm)			
Treatments	*Melilotus indica* L.	*Anagallis arvesis* L.	*Phalaris minor* L.	*Fumaria indica* L.
T_1_	66.8 a	28.7 a	94.7 a	55.6 a
T_2_	29.5 ab	5.6 ab	83.2 a	22.3 bc
T_3_	26.7 b	6.2 ab	67.6 a	23.3 b
T_4_	28.4 b	6.7 ab	86.7 a	23.6 b
T_5_	24.9 b	5.0 ab	64.5 a	17.4 cd
T_6_	25.6 b	7.1 ab	62.4 a	17.6 cd
T_7_	26.0 b	23.0 ab	72.1 a	16.6 d
T_8_	29.0 b	3.2 ab	64.5 a	16.3 d
T_9_	20.0 b	1.5 c	46.9 a	17.4 cd
T_10_	34.6 ab	9.5 ab	61.3 a	17.1 cd
HSD (0.05)	17.34	25.66	73.65	5.49

*Means sharing the same letter in a column did not differ from each other at a 5% (0.05) probability level. T_1_, weedy check; T_2_, metribuzin at 175 g a.i ha^–1^; T_3_, metribuzin at 140 g a.i ha^–1^; T_4_, metribuzin at 105 g a.i ha^–1^; T_5_, metribuzin at 175 g a.i ha^–1^ + Ad-500 at 400 ml ha^–1^; T_6_, metribuzin at 140 g a.i ha^–1^ + Ad-500 at 400 ml ha^–1^; T_7_, metribuzin at 105 g a.i ha^–1^ + Ad-500 at 400 ml ha^–1^; T_8_, metribuzin at 175 g a.i ha^–1^ + Bio-power at 400 ml ha^–1^; T_9_, metribuzin at 140 g a.i ha^–1^ + Bio-power at 400 ml ha^–1^; T_10_, metribuzin at 105 g a.i ha^–1^ + Bio-power at 400 ml ha^–1^.*

### Physiological Parameters of Weeds

**FIGURE 2:**
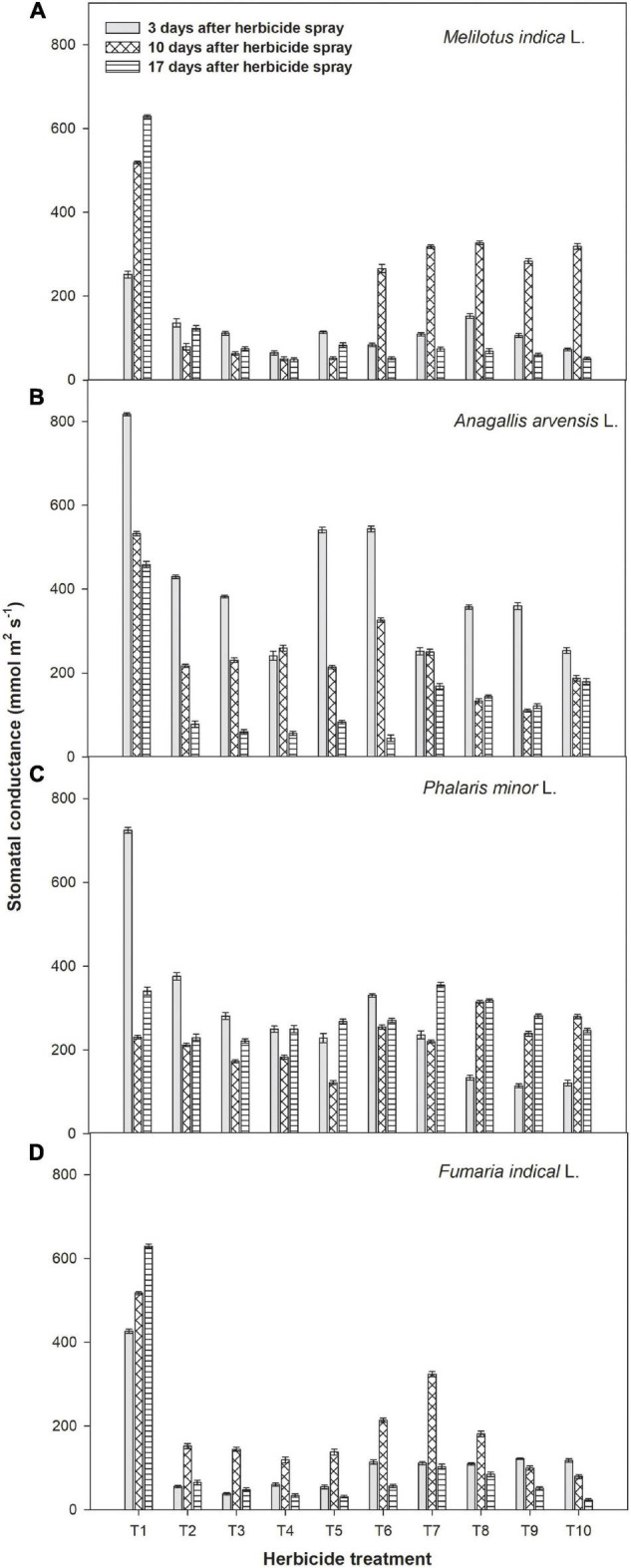
Impact of metribuzin with and without adjuvants on net photosynthetic rate of *Melilotus indica* L. **(A)**, *Anagallis arvensis* L. **(B)**, *Phalaris minor* L. **(C)**, and *Fumaria indica* L. **(D)** at 3, 10, and 17 days after herbicide spray (average of 2 years’ data). T_1_, weedy check; T_2_, metribuzin at 175 g a.i. ha^–1^; T_3_, metribuzin at 140 g a.i. ha^–1^; T_4_, metribuzin at 105 g a.i. ha^–1^; T_5_, metribuzin at 175 g a.i. ha^–1^ + Ad-500 at 400 ml ha^–1^; T_6_, metribuzin at 140 g a.i. ha^–1^ + Ad-500 at 400 ml ha^–1^; T_7_, metribuzin at 105 g a.i. ha^–1^ + Ad-500 at 400 ml ha^–1^; T_8_, metribuzin at 175 g a.i. ha^–1^ + Bio-power at 400 ml ha^–1^; T_9_, Metribuzin at 140 g a.i. ha^–1^ + Bio-power at 400 ml ha^–1^; T_10_, metribuzin at 105 g a.i. ha^–1^ + Bio-power at 400 ml ha^–1^.

Gas exchange parameters of the four weeds were recorded at 3, 10, and 17 days after metribuzin application. Metribuzin alone or with adjuvants resulted in the lowest rate of photosynthesis of weeds than weedy check when measured at different times (Figure 2). After 17 days of herbicides spray, net photosynthetic rates of *A. arvensis*, *P. minor*, *F. indica*, and *M. indica* were significantly reduced by 0.30, 0.51, 0.29, and 0.45 μmol m^2^ s^–1^, respectively, in plots where metribuzin at 175 g a.i ha^–1^ + Bio-power at 400 ml ha^–1^ were sprayed, which was followed by metribuzin at 175 g a.i ha^–1^ + Ad-500 at 400 ml ha^–1^.

**FIGURE 3:**
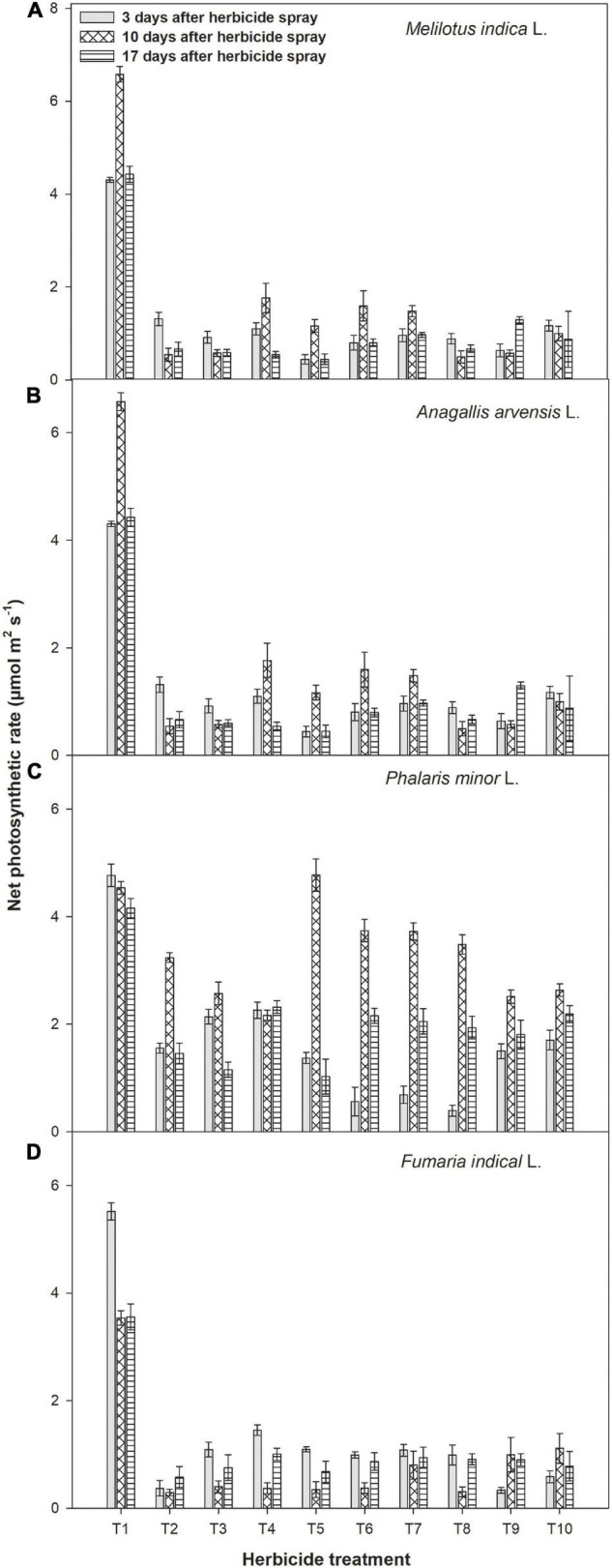
Impact of metribuzin with and without adjuvants on transpiration rate of *Melilotus indica* L. **(A)**, *Anagallis arvensis* L. **(B)**, *Phalaris minor* L. **(C)**, and *Fumaria indica* L. **(D)** at 3, 10, and 17 days after herbicide spray (average of 2 years’ data). T_1_, weedy check; T_2_, metribuzin at 175 g a.i. ha^–1^; T_3_, metribuzin at 140 g a.i. ha^–1^; T_4_, metribuzin at 105 g a.i. ha^–1^; T_5_, metribuzin at 175 g a.i. ha^–1^ + Ad-500 at 400 ml ha^–1^; T_6_, metribuzin at 140 g a.i. ha^–1^ + Ad-500 at 400 ml ha^–1^; T_7_, metribuzin at 105 g a.i. ha^–1^ + Ad-500 at 400 ml ha^–1^; T_8_, metribuzin at 175 g a.i. ha^–1^ + Bio-power at 400 ml ha^–1^; T_9_, metribuzin at 140 g a.i. ha^–1^ + Bio-power at 400 ml ha^–1^; T_10_, metribuzin at 105 g a.i. ha^–1^ + Bio-power at 400 ml ha^–1^.

Foliar applied metribuzin with or without adjuvants significantly lowered the transpiration rate of *F. indica*, *M. indica*, *A. arvensis*, and *P. minor* (Figure 3). However, the highest transpiration rate was recorded in the control treatment when compared with the herbicide-treated plots. In *M. indica*, transpiration rate was minimal with metribuzin at 175 g a.i ha^–1^ + Ad-500 at 400 ml ha^–1^ at three stages of observation.

**FIGURE 4:**
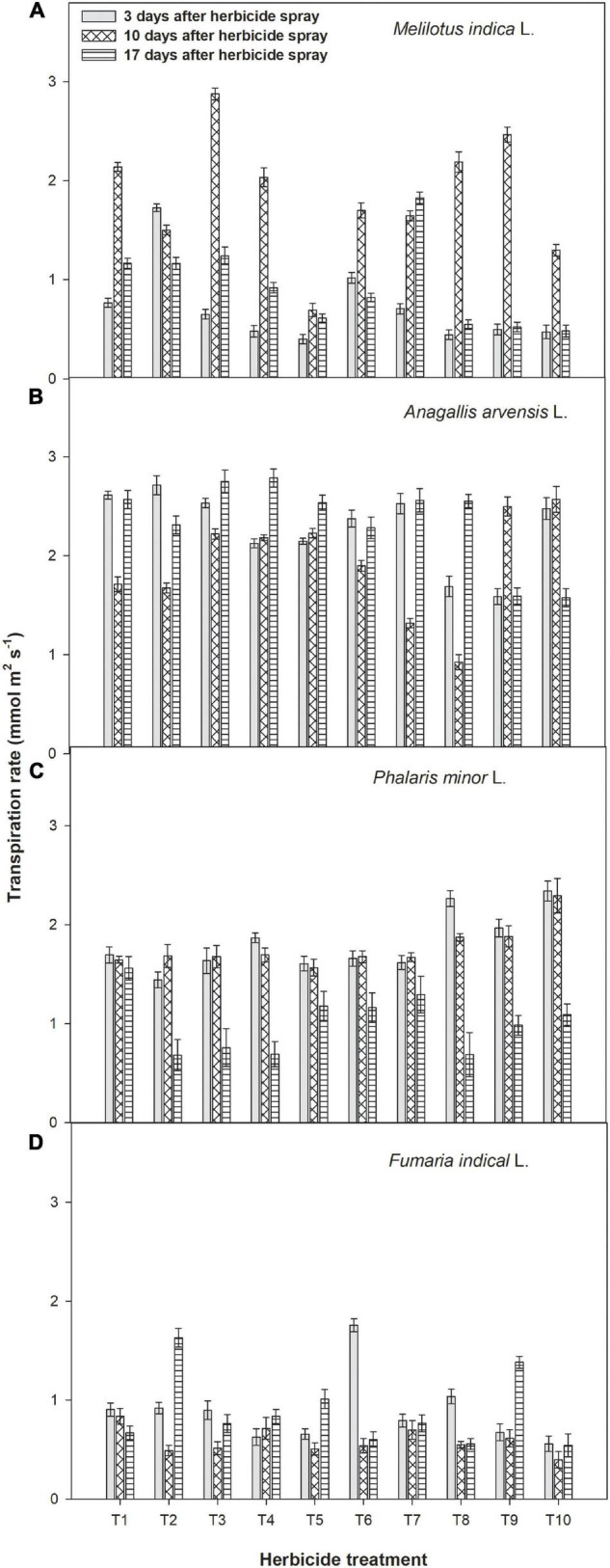
Impact of metribuzin with and without adjuvants on stomatal conductance of *Melilotus indica* L. **(A)**, *Anagallis arvensis* L. **(B)**, *Phalaris minor* L. **(C)**, and *Fumaria indica* L. **(D)** at 3, 10, and 17 days after herbicide spray (average of 2 years’ data). T_1_, weedy check; T_2_, metribuzin at 175 g a.i. ha^–1^; T_3_, metribuzin at 140 g a.i. ha^–1^; T_4_, metribuzin at 105 g a.i. ha^–1^; T_5_, metribuzin at 175 g a.i. ha^–1^ + Ad-500 at 400 ml ha^–1^; T_6_, metribuzin at 140 g a.i. ha^–1^ + Ad-500 at 400 ml ha^–1^; T_7_, metribuzin at 105 g a.i. ha^–1^ + Ad-500 at 400 ml ha^–1^; T_8_, Metribuzin at 175 g a.i. ha^–1^ + Bio-power at 400 ml ha^–1^; T_9_, metribuzin at 140 g a.i. ha^–1^ + Bio-power at 400 ml ha^–1^; T_10_, metribuzin at 105 g a.i. ha^–1^ + Bio-power at 400 ml ha^–1^.

Metribuzin applied at 175 g a.i ha^–1^ + Ad-500 at 400 ml ha^–1^ showed minimum transpiration rate in *A. arvensis*, *P. minor*, and *F. indica* when recorded at 17 days after herbicide spray. Stomatal conductance was found maximal in control treatment as three stages of observations (Figure 4). Data showed that spray of metribuzin with or without adjuvants reduced the stomatal conductance of all weeds after 3, 10, and 17 days of application. Application of metribuzin at 175 g a.i ha^–1^ with both adjuvants each at 400 ml ha^–1^ showed minimum stomatal conductance in *M. indica* at either reading time. In the case of *A. arvensis*, *P. minor*, and *F. indica*, the lowest stomatal conductance was recorded with metribuzin at 175 g a.i ha^–1^ + Bio-power at 400 ml ha^–1^ followed by metribuzin at 175 g a.i ha^–1^ + Ad-500 at 400 ml ha^–1^ (Figure 4).

## Discussion

*Phalaris minor*, *M. indica*, *F. indica*, and *A. arvensis* are weeds of winter wheat and cause a severe reduction in wheat yield if not controlled at the appropriate stage (Abbas et al., 2018b; Javaid et al., 2020). However, an effective dose, optimum time, and suitable mixture of herbicide along with adjuvants is required to suppress the weed growth and enhance wheat yield (Javaid and Tanveer, 2013; Safdar et al., 2021). In our experiment, highest and lowest spike and non-spike-bearing tillers were obtained in plots that showed maximum weeds control. This might be the addition of adjuvants that improved weed control efficiency of metribuzin and provided more resources to crop plants instead of weeds. According to Rizwan et al. (2018), the application of herbicide increases the number of spike-bearing tillers (m^–2^) significantly. Our findings are further supported by Moraes et al. (2021) who stated that herbicides applied with adjuvants either at full or reduced doses significantly decreased the weeds infestation than herbicides used alone and resultantly improved the spike-bearing tillers with concomitant reduction of non-spike-bearing tillers by supplying more space, light, and nutrients to crop plants. Further studies suggested that optimum weed control was based on the selection of herbicide and adjuvants along with weed-specific situations (Javaid et al., 2012; Moraes et al., 2021). Similarly, Khalil et al. (2010) also revealed that greater numbers of spike-bearing tillers were obtained with the application of carfentrazone-ethyl + isoproturon as compared with weedy check treatment. The increment in spike length, the height of the plant, and grains per spike with all treatments of metribuzin over the weedy check might have been due to lower weed-crop competition for available resources that enhanced the water and nutrient use efficiency of wheat crop (Javaid et al., 2016, 2020; Shah et al., 2018). Our findings suggested that the addition of Bio-power enhances the efficacy of metribuzin even at lower doses (105 or 140 g a.i ha^–1^) in terms of spike length, the height of the plant, and grains per spike in comparison with Ad-500 or without adjuvant treatments. The greater grains per spike resulted in metribuzin + adjuvant-treated plots were due to the least competition of weeds and maximum photosynthates produced by the wheat plant (Javaid et al., 2020). According to Ashrafi et al. (2009), the application of isoproturon, triasulfuron, and bromoxynil + MCPA expressively enhanced the yield constituents of wheat over weedy check. Similar findings are also described by Zangoueinejad et al. (2018) who stated that the application of metribuzin showed maximum plant height of tomato with maximum weed control. Our conclusions are braced by Colbach et al. (2019) who declared that application of herbicide after the emergence of weeds affected the crop plant height and leads to the trade-off between parameters driving potential crop production and minimized weed-inflicted yield losses. Singh et al. (2019) also detected that the length of wheat spike decreased when the density of weeds was higher. They also predicted that the length of wheat spike decreased by 10% when wild oat density augmented up to 2.6 plants m^–2^. Cheema et al. (2006) also stated that a mixture of herbicides with adjuvants produced greater grains per spike than herbicides used alone. In our experiment, 1,000-grain weight, biological yield, and grain yield were increased with the full dose of postemergence application of metribuzin added with both adjuvants (Bio-power and Ad-500). This might be due to the availability of more resources to crop plants that resulted in the suppression of weed growth and improved protein synthesis and more transportation of photosynthates from source to sink. These fallouts are in promise with the findings by Bibi et al. (2008) who exposed that herbicides have substantial impacts on wheat of 1,000-grain weight. Similarly, Javaid et al. (2020) also stated that when isoproturon was applied as a foliar spray, the highest biological yield was obtained. Hamouda et al. (2021) concluded that the addition of Argal or Techno oil to herbicides spray solutions at reduced rates, especially at three-fourths of the recommended rate resulted in enhancing the herbicidal activity against weeds and increasing wheat grain yield. This might be due to the maximum weed control in wheat along with no injury caused by the use of this herbicide quantity and adjuvant. These findings were supported with the study of Abbas et al. (2018a) who described that if the herbicide is applied in combination with adjuvants, it will control weeds better and ultimately increase the grain yield.

At three stages of observations, maximum physiological characteristics of wheat-like net stomatal conductance photosynthetic rate and transpiration rate were measured in the weedy check than all other herbicide applications. A full dose of metribuzin with both adjuvants (Ad-500 and Bio-power) each at 400 ml ha^–1^ produces the highest gaseous exchange parameters after 3 days of herbicide spray at an earlier stage indicating that its application imparted litter bit impact on the wheat crop as compared with lower doses of metribuzin. The metribuzin without adjuvants even at the highest dose showed less weed control and yield of the wheat crop when compared with added adjuvants. Our data showed that gas exchange parameters of the wheat crops were also affected with metribuzin treatment compared with the weedy check when recorded at 3 days after herbicide spray. However, these parameters were significantly improved at the lateral stage (e.g., 10 and 17 days after spray). Todorova et al. (2022) treated wheat plants with herbicide Serrate^®^ (Syngenta) and were subsequently subjected to drought or flooding stress for 7 days. They concluded that herbicide pretreatment did not cause significant alterations in photosynthesis and fluorescence parameters in alone- or combined-treated seedlings. A significant reduction in gas exchange parameters (net photosynthesis rate, stomatal conductance, transpiration rate, and water use efficiency) values during drought or flooding was observed. The mixing of adjuvants with metribuzin boosted its efficacy in terms of weed control, which compensates for the harmful effects of metribuzin on gas exchange parameters of wheat by reducing the resources competition between weeds and wheat. According to Naseer-ud-din et al. (2011), Vandoorne et al. (2012), and Shukla et al. (2015), herbicides affect photosynthetic activities by disrupting stomatal control of the CO_2_ supply, carbon reduction cycle, chlorophyll activity, and thylakoid electron transport. These results are also in harmony with Jerzykiewicz and Klobus (2007) who checked the effect of triazines and phenyl ureas on photosynthetic activity in cucumber leaves. They found that triazines and phenyl ureas stopped the activity of photosynthesis in the cucumber plant by binding certain sites within the plant’s photosystem II. The triangles and phenylases in the QB site bind to the D1 protein in PS-II and inhibit the transfer of the electron between the primary receptor of the electrons Q and plastoquinone. In our findings, fresh and dry weight of weeds and the number of weeds at 21 days after herbicide spray and at harvest reduced significantly with all treatments of herbicide than weedy check. However, the highest weeds suppression, least fresh, and dry weight of weeds were observed with the full dose of metribuzin added with Bio-power or Ad-500. It was probably due to the addition of an adjuvant, which improves the efficiency of metribuzin. The study by Harmoney et al. (2007) which also supported our findings revealed that imazapyr and glyphosate inhibited the growth of weeds and led to the stunted plants of weeds. These findings are in harmony with those of Zakariyya et al. (2013) who observed operative control and lower fresh and dry biomass of weeds with Puma super and Buctril super applications. Shah et al. (2017) depicted that the application of 50% clodinafop + 50% bromoxynil at tillering time resulted in higher grain yield and less weeds number and biomass. Zand et al. (2007) also reported that after the emergence of weeds, the application of bromocycynyl + MCPA reduced the dry weight of weeds significantly and improved the yield of wheat.

Gas exchange parameters of all four weeds were measured at three stages after herbicide spray and noted a significant reduction over the weedy check. According to the study of Agostinetto et al. (2016), photosynthetic rate, stomatal conductance, and transpiration of wheat weeds were negatively affected by metribuzin, metsulfuron, and 2,4-D herbicides at 24 and 120 h after spraying compared with control. Among all the treatments, a full dose of metribuzin with both adjuvants caused a maximum reduction in stomatal conductance, net photosynthetic rate, and transpiration rate of all four weeds at 17 days after herbicide spray. The decline in the above traits due to metribuzin was probably due to its negative impact on photosystem II (PS-II) because after application it is transported through the xylem. It disrupts the transmission electron between the primary and secondary receptors of the PS-II (Monaco et al., 2002). Similarly, Huang et al. (2012) observed that various concentrations of glyphosate (0%, 0.3%, 0.5%, 1.0%, and 2.0%) decreased the net photosynthetic rate, physicochemical properties, and the relative rate of electron transport through PS-II of cogon grass (*Imperata cylindrical* L.).

## Conclusion

The use of metribuzin at 175 g a.i ha^–1^ + Bio-power at 400 ml ha^–1^ was more effective in reducing photosynthetic parameters of weeds (*A. arvensis*, *M. indica*, *P. minor*, and *F. indica*) compared with all other herbicide alone or with adjuvant treatments. So the application of metribuzin with tank mixing of Bio-power proved most effective in controlling tested weeds of wheat. Moreover, the maximum wheat growth and yield was also achieved with this treatment, indicating that adjuvant “Bio-power” is the most suitable combination with metribuzin that has no toxic effects on wheat crop.

## Data Availability Statement

The original contributions presented in the study are included in the article/supplementary material, further inquiries can be directed to the corresponding authors.

## Author Contributions

MMJ conceived the idea and conducted the research. MINB, HW, and AM carried out the investigation. AA, MAN, and AM helped in data analysis and collection of literature review. AM, KA, NK, AA-D, XW, and SF provided with technical expertise to streamline the findings. MINB and HW helped in the writing of the original draft. All authors carefully read, revised, and approved the article for submission.

## Conflict of Interest

The authors declare that the research was conducted in the absence of any commercial or financial relationships that could be construed as a potential conflict of interest.

## Publisher’s Note

All claims expressed in this article are solely those of the authors and do not necessarily represent those of their affiliated organizations, or those of the publisher, the editors and the reviewers. Any product that may be evaluated in this article, or claim that may be made by its manufacturer, is not guaranteed or endorsed by the publisher.
